# Comment on: “Neural Correlates of Balance Skill Learning in Young and Older Individuals: A Systematic Review and Meta-Analysis”

**DOI:** 10.1186/s40798-025-00814-z

**Published:** 2025-01-26

**Authors:** Thierry Paillard, Frédéric Noé

**Affiliations:** Université de Pau et des Pays de l’Adour, E2S, MEPS, ZA Bastillac Sud, 11 rue Morane Saulnier, Tarbes, 65000 France

Dear Editor,

We read with great interest the very interesting systematic review and meta-analysis by Bakker et al. [1] about the neural correlates of balance skills learning in young and older individuals. It is clearly rich in information and useful for sport trainers to optimize motor performance and to prevent injuries among athletes, and help therapists to enhance the effects of their therapeutic programs as well as their preventive measures against falls in frail or older subjects.

Their article [1] raises fundamental questions relating to the design of balance training protocols (nature of postural tasks and number of training sessions to be planned) and to the behavioral (balance skills) and neural (spinal excitability, corticospinal excitability, cortical activity, brain outcomes, neurochemical blood markers of neural adaptation) adaptations expected. As previously suggested [2], Bakker et al. [1] underlined that the improvements resulting from learning on a specific postural task generated little or no transfer to other postural tasks. Hence, the balance training methods classically implemented involving postural tasks with varying degrees of difficulty and instability based on different postures and support conditions would be of little use compared to specific postural tasks linked to the individual’s physical practice. Bakker et al. [1] also assumed that balance skill learning and the accompanying neural adaptations occur rapidly and independently of age, with little to no dependence on training dose (i.e., number of training sessions) or correspondence between behavioral and neural adaptations. According to these authors, if balance skills are developed as much (or almost as much) after 1 to 3 training sessions as after more than 3 sessions (additional sessions), there is no need to do more than 3 training sessions to develop balance skills [1].

This raises the question of the trainability of balance skills (i.e., the possibility of improving them through balance training independently of the baseline level) and the difficulty of improving them through long-term training. Nevertheless, studies about the influence of sport expertise and balance skills have shown that athletes displayed better balance skills than non-athletes and that the most successful athletes in term of sport competition level have the best balance skills [3]. These results suggest that balance skills could be improved by long-term sport training, challenging the idea that balance skills could plateau after a few training sessions. It is also known that some athletes display better balance skills than others after the same duration of training/practice [3], thus suggesting that the trainability of balance skills depends more on individual natural predispositions than on the number of training sessions. Without mentioning trainability, natural predispositions could directly influence balance skills, since proprioception, which strongly influences balance skills, is more accurate in elite athletes than in sub-elite athletes independently of the amount of training [4].

The trainability of balance skills would be low and highly specific to postural tasks trained [1]. Nevertheless, the influence on long-term sporting/motor experience and individual natural predispositions should be further investigated to improve our understanding of the mechanisms underlying the learning of balance skills.

## Data Availability

Not applicable.
